# The sirtuins, oxidative stress and aging: an emerging link

**DOI:** 10.18632/aging.100544

**Published:** 2013-03-07

**Authors:** Philip I. Merksamer, Yufei Liu, Wenjuan He, Matthew D. Hirschey, Danica Chen, Eric Verdin

**Affiliations:** ^1^ Gladstone Institute of Virology and Immunology, University of California, San Francisco, CA 94158, USA; ^2^ Department of Molecular and Cell Biology, University of California, Berkeley, CA 94720; USA; ^3^ Program in Metabolic Biology, Nutritional Sciences and Toxicology, University of California, Berkeley, CA 94720, USA

**Keywords:** sirtuins, SIRT1, SIRT3, oxidative stress, mitohormesis, acetylation

## Abstract

Reactive oxygen species (ROS) are a family of compounds that can oxidatively damage cellular macromolecules and may influence lifespan. Sirtuins are a conserved family of nicotinamide adenine dinucleotide (NAD+)-dependent protein deacetylases that regulate lifespan in many model organisms including yeast and mice. Recent work suggests that sirtuins can modulate ROS levels notably during a dietary regimen known as calorie restriction which enhances lifespan for several organisms. Although both sirtuins and ROS have been implicated in the aging process, their precise roles remain unknown. In this review, we summarize current thinking about the oxidative stress theory of aging, discuss some of the compelling data linking the sirtuins to ROS and aging, and propose a conceptual model placing the sirtuins into an ROS-driven mitochondria-mediated hormetic response.

## Reactive oxygen species

ROS are a group of compounds derived from the incomplete reduction of molecular oxygen. They include—but are not limited to—the superoxide anion (O_2_*^−^), hydrogen peroxide (H_2_O_2_), and the hydroxyl radical (OH*). While ROS are generated through a variety of mechanisms, their primary physiological source is cellular respiration. During respiration, electrons are passed through four protein complexes (Complex I, II, III, and IV) that reside in the mitochondrial inner membrane. The majority of electrons proceed through each complex and ultimately to molecular oxygen, which is reduced to water. However, a small percentage of electrons can escape the electron transport chain prematurely, leading to incomplete reduction of molecular oxygen and formation of the superoxide anion O_2_*^−^[1]. The anionic nature of O_2_*^−^ restricts both its ability to diffuse throughout the cell and its reactivity toward electron-rich substrates. O_2_*^−^ primarily reacts with and inactivates enzymes that contain Fe-S clusters, such as dehydratases and aconitase [2]. O_2_*^−^ is detoxified by dismutation into H_2_O_2_ and O_2_ in a process that occurs spontaneously but is rapidly accelerated by superoxide dismutase (SOD) [3].

**Figure 1:**
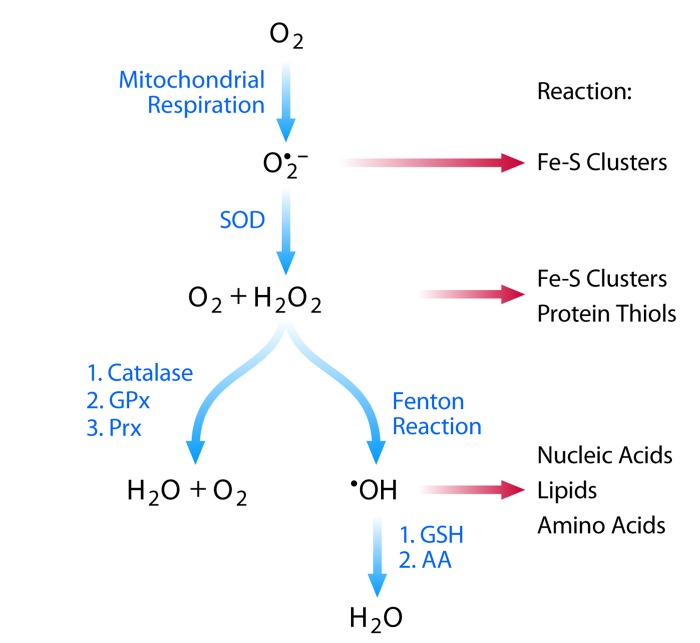
Reactive oxygen species: production and protection Schematic showing the major reactive oxygen species associated with cellular respiration. Blue arrows indicate detoxification mechanisms while red arrows indicate reactivity for each ROS.

Other ROS include hydrogen peroxide (H_2_O_2_) and the hydroxyl radical (OH*). While H_2_O_2_ can rapidly diffuse throughout the cell, its reactivity is restricted to proteins containing transition metals (such as Fe-S clusters) or low pKa thiols [4]. The transition metal-catalyzed reduction of H_2_O_2_ to OH*, however, is highly reactive. OH* displays the broadest reactivity and indiscriminately oxidizes lipids, nucleic acids, and amino acids (Figure 1).

Cells have numerous enzymatic mechanisms to detoxify H_2_O_2_, including catalase, glutathione peroxidase, and the peroxiredoxins, which reduce H_2_O_2_ to H_2_O and O_2_. OH* is primarily detoxified by small-molecule reductants, such as glutathione and ascorbate. When these detoxification mechanisms are insufficient to neutralize the ROS, cellular macromolecules may become oxidatively damaged, a state defined as oxidative stress.

It has long been appreciated that oxidative damage increases during aging and that caloric restriction—which has been postulated to mitigate such damage—can increase the lifespan of several model organisms [5]. While the mechanisms governing caloric restriction-mediated lifespan extension and oxidative stress resistance remain incompletely understood, the sirtuin family of proteins has attracted considerable attention for regulating these phenotypes. Here, we discuss data linking the sirtuins to the oxidative stress response, caloric restriction, and longevity.

## The sirtuins

Sir2 (silent information regulator 2) from *Sacchromyces cerevisiae* and the conserved mammalian orthologs, collectively called sirtuins, are NAD+-dependent histone/protein deacetylases. Sirtuins catalyze the removal of acetyl groups from the side chain amino group of lysine residues. This reaction consumes NAD+ and generates nicotinamide (NAM) and 2’-*O*-acetyl-ADP-ribose [6]. Seven sirtuin members have been identified in mammals, named numerically as SIRT1-7. They have distinct subcellular localizations: SIRT1, 6, and 7 are found in the nucleus, SIRT2 is cytosolic, and SIRT3, 4, and 5 are primarily located in the mitochondria [7]. While SIRT1, 2, and 3 have robust deacetylase activities, the other sirtuins exhibit weak or no deacetylase activity. Recently, SIRT5 was found to catalyze the removal of malonyl and succinyl groups from lysines suggesting that the remaining sirtuins may target additional modes of lysine acylation [8, 9].

## Sir2 and oxidative stress

Much of the early evidence linking the sirtuins to oxidative stress was obtained by studying the effect of genetic manipulation of Sir2 on aging in eukaryotic model organisms such as *S. cerevisiae*, *C. elegans*, and *D. melanogaster*. Disruption of the *SIR2* gene severely shortens lifespan in *Saccharomyces cerevisiae*, while its overexpression increases lifespan beyond that for wild-type cells [10]. Likewise, overexpression of Sir-2.1, the homolog of yeast Sir2, increases lifespan in *C. elegans* [11] and Drosophila [12]. Intriguingly, the lifespan extension observed during caloric restriction in yeast is abrogated when Sir2 is deleted begging the question that Sir2 may influence lifespan by diminishing oxidative stress [13]. In support of this notion, overexpression of Sir2 rescues the shortened lifespan phenotype observed when *S. cerevisiae* are treated with hydrogen peroxide [14]. Moreover, Sir2 regulates the asymmetric segregation of oxidatively damaged proteins from daughter cells to mother cells during cell division in yeast providing a putative mechanistic basis for Sir2's role in oxidative stress resistance and lifespan extension [15]. The role of Sir2 in mediating lifespan extension, however, has been recently called into question with new experiments demonstrating that Sir2 overexpression does not enhance longevity in *C. elegans* or *Drosophila*[16]. Clearly, more work is required to resolve these conflicting data and to determine the relative importance of sirtuins for longevity in these metazoans.

## The mammalian sirtuins and oxidative stress

**Figure 2:**
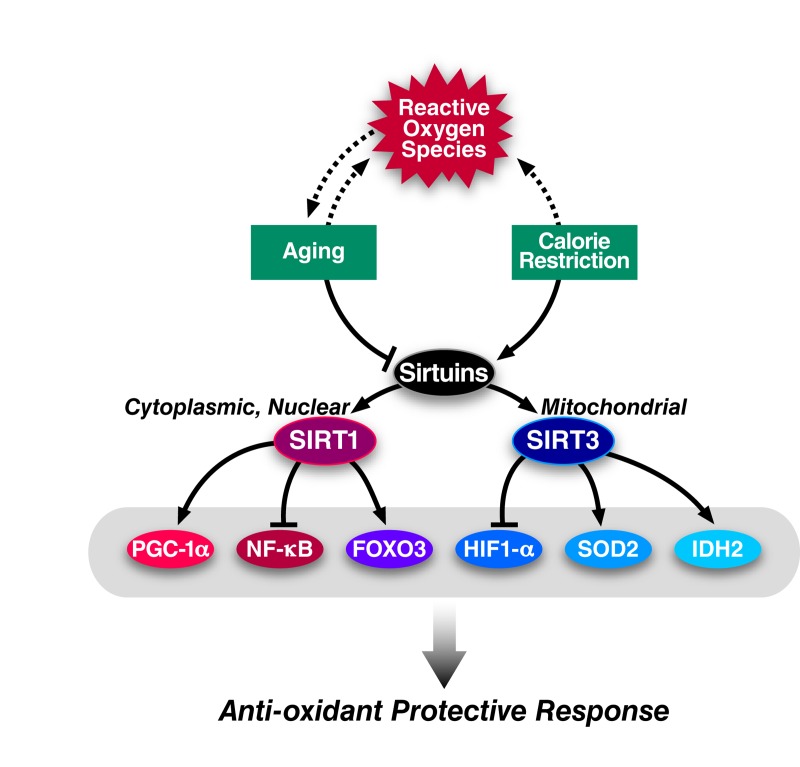
Regulation of ROS by sirtuins Diagram showing how ROS, aging, and caloric restriction interact to influence the activity of the cytoplasmic/nuclear sirtuin, SIRT1, and the mitochondrial sirtuin, SIRT3. Upon activation, SIRT1 and SIRT3 deacetylate several proteins that promote resistance to oxidative stress. Arrows indicate positive regulation while hash-marks indicate negative regulation. Solid lines reflect robust experiment evidence for an interaction while dashed lines indicate putative interactions.

The two closest mammalian homologs of yeast Sir2 are the nuclear-localized SIRT1 and the mitochondrion-localized SIRT3 (see Figure 2 for an overview). Strong evidence supports a role for SIRT1 mediating an oxidative stress response by directly deacetylating several transcription factors that regulate antioxidant genes. Notably, SIRT1 activates several members of the FOXO family of transcription factors which promote the expression of stress response genes including SOD2 [17-19]. For example, SIRT1 functions in an autoregulatory loop along with the early growth response protein ERG1 to regulate SOD2 to protect contracting muscle cells from oxidative stress [20, 21].

SIRT1 also promotes mitochondrial biogenesis by activating peroxisome proliferator-activated receptor co-activator 1-α (PGC-1α) [22]. PGC-1α increases mitochondrial mass and upregulates the expression of oxidative stress genes including glutathione peroxidase (GPx1), catalase, and manganese SOD (MnSOD) [23]. Finally, SIRT1 inactivates the p65 subunit of NF-ĸB through direct deacetylation. NF-ĸB inhibition suppresses the inducible nitric oxide synthase (iNOS) and nitrous oxide production and thus may lower the cellular ROS load [24]. Given its role in antioxidant response, whether SIRT1 activation contributes to CR mediated lifespan extension has been extensively studied. CR fails to increase the lifespan of SIRT1 knock-out mice, and these mice do not increase their physical activity, a phenotype typically associated with calorically-restricted mice [25, 26]. Similarly, SIRT1 overexpression mimics a caloric restriction phenotype [27]. Precisely how SIRT1 functions during CR remains an open question, but emerging evidence suggests that p53 plays an important role in modulating SIRT1 during CR [28].

Mitochondria account for the majority of cellular ROS production. Mitochondrial SIRT3 deacetylates and activates several enzymes that are critical in maintaining cellular ROS levels. SIRT3 deacetylates SOD2 at two important lysine residues to boost its catalytic activity and the catalytic activity of SOD2 is diminished when SIRT3 is deleted [29]. SIRT3 knock-out mice fail to reduce their levels of lipid peroxidation and protein carbonylation that are typically observed during caloric restriction indicating that SIRT3 is necessary for caloric restriction to mitigate oxidative stress. Additionally, SIRT3 stimulates the activity of mitochondrial isocitrate dehydrogenase, IDH2, during caloric restriction through direct deacetylation [30]. IDH2 promotes the conversation of NADP^+^ to NADPH which in turn provides the reducing equivalents for conversion of oxidized to reduced glutathione. In support of this biochemical data, SIRT3 is required to protect calorically-restricted mice from age-associated hearing loss [30, 31].

Another link between SIRT3 and oxidative stress comes from the field of oncology. Since ROS can severely damage nucleic acids, it is not surprising that oxidative stress can promote tumorigenesis. SIRT3 knock-out mouse embryonic fibroblasts (MEFs) exhibit higher ROS levels, greater genomic instability, and increased sensitivity to oncogenic transformation compared to wild-type fibroblasts (Kim et al, 2010). Intriguingly, overexpression of SOD2 suppresses oncogenic transformation in SIRT3 knock-out MEFs suggesting that SIRT3 may protect against tumorigenesis through an oxidative stress mechanism. In support of the above in vitro data, mice deficient for SIRT3 are more susceptible to cancer and many human tumors display reduced SIRT3 levels compared to healthy tissues [32]. In addition to suppressing the formation of cancer, SIRT3 can also combat established tumors. Overexpression of SIRT3 suppresses tumor proliferation via inhibiting the activity of the hypoxia inducible factor-1α (HIF-1α) [33, 34]. Mechanistically, the HIF-1α protein is stabilized by the presence of ROS and activates a gene expression program that enhances survival and growth in hypoxic environments, as typically found in solid tumors. Given HIF-1α is activated via a ROS-mediated mechanism it is likely that SIRT3 decreases HIF-1α activity by suppression of ROS levels.

## Other sirtuins

Besides SIRT1 and SIRT3, other sirtuins also contribute to the cellular response to oxidative stress. SIRT2 deacetylates FOXO3a and promotes cellular resistance to H_2_O_2_[35], similar to SIRT1 regulation of oxidative stress via FOXO family members. SIRT6 deficient cells display sensitivity to oxidative stress and a reduced capacity for DNA repair, while SIRT6 knockout mice show many hallmarks of premature aging [36]. In support of the anti-aging effects of SIRT6, male mice overexpressing SIRT6 have a significantly longer lifespan than their wild-type counterparts [37]. Mechanistically, SIRT6 mono-ADP ribosylates poly (ADP-ribose) polymerase 1 (PARP1) to stimulate DNA double-strand break (DSB) repair in response to oxidative stress [38, 39]. Mutations in several DSB repair genes are often associated with premature aging which could explain in part why SIRT6 mutants also have many characteristics of premature aging [40]. Lastly, SIRT7 has been implicated in oxidative stress resistance through an investigation of primary cardiomyocytes from SIRT7 knock-out mice. These cells are increasingly sensitive to both genotoxic and oxidative insults such as doxorubicin and H_2_O_2_ compared to wild-type. [41]. Together these studies reflect the importance of the sirtuin family in oxidative stress and will hopefully spur future studies to carefully decipher their mechanisms.

## Mitohormesis and mitochondrial hyperacetylation

In 1956, Harman proposed that oxidative stress may cause the observed physiological decline in cellular and organismal functions that occur during aging [42]. This theory has since been supported by studies showing that cellular oxidative damage increases with aging and that overexpression of some anti-oxidant genes increase lifespan in *Drosophila*[5]. However, transgenic mice over-expressing related anti-oxidant genes do not live longer than their wild-type counterparts raising some doubts on the universal relevance of this theory as a mechanism of aging[43]. These conflicting data suggest a more complex mode of regulation.

**Figure 3:**
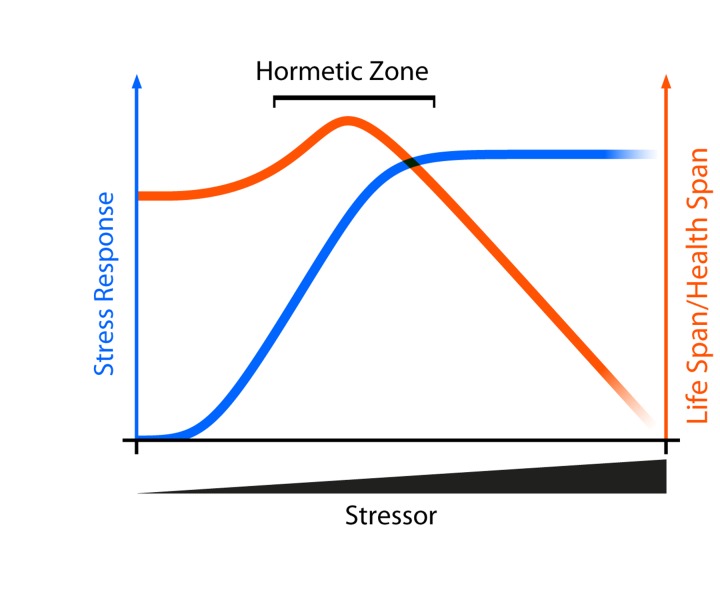
Aging and Mitohormesis Theoretical curve showing how low doses of a stressor may have beneficial effects by activating intracellular stress response pathways. If the stressor exceeds the capacity of the stress response system to maintain homeostasis, then deleterious phenotypes are observed.

Mitohormesis may reconcile many of the seemingly conflicting data relating to the role of oxidative stress and health. First coined by Ristow and colleagues, mitohormesis is an application of the theory of hormesis in which a stressor may have beneficial effects at relatively low doses and deleterious effects at high doses [44]. Conceptually, small and/or transient amounts of reactive oxygen species elicit a protective stress response that may improve lifespan. Relatively large and/or chronic amounts of the same species, however, cause cellular damage or death because they exceed the capacity of the oxidative stress response to maintain homeostasis (Figure 3). In support of this notion, low-doses of paraquat, which induce O_2_-* formation, increase lifespan in *Caenorhabditis elegans* [45], while high-doses of paraquat reduce lifespan [46]. Mitohormesis may also explain how caloric restriction extends lifespan. Caloric restriction was originally thought to decrease the production of ROS by dampening mitochondrial respiration. However, mitochondrial respiration actually increases during caloric restriction in yeast, worms, and mice which may in turn increase ROS associated with respiration [47-49]. A moderate ROS increase during caloric restriction can stimulate oxidative stress resistance mechanisms which can minimize oxidative cellular damage over time, delay age-associated phenotypes, and extend lifespan [44, 50]. While attractive, the mechanistic link between metabolic stressors such as caloric restriction and mitohormesis remain unresolved.

**Figure 4:**
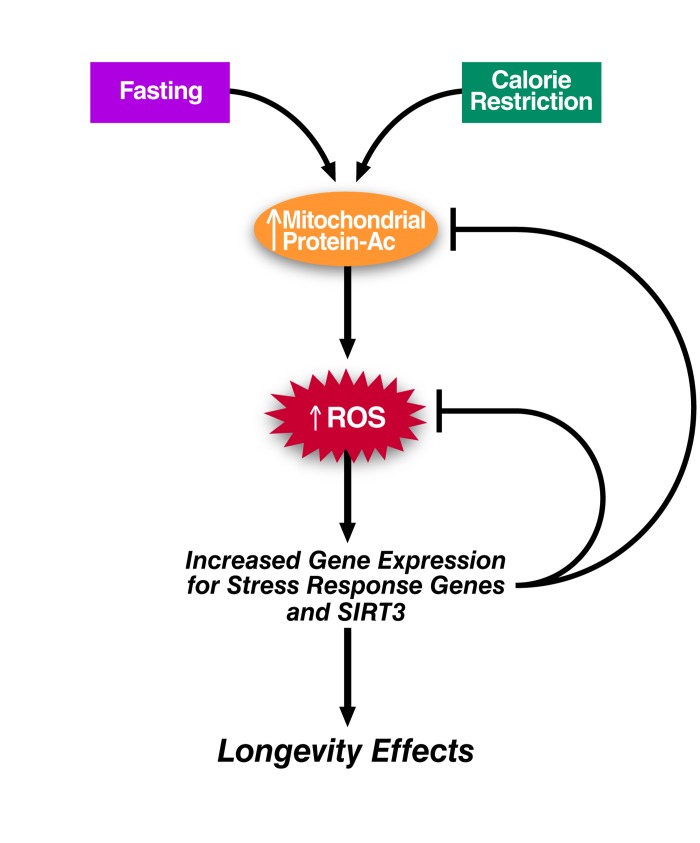
Hyperacetylation and Mitohormesis Theoretical model linking mitochondrial hyperacetylation to the generation of ROS and mitohormesis.

It is noteworthy that during caloric restriction, global mitochondrial protein acetylation increases in metabolically active tissue such as liver [51]. In fact, several physiological stresses including fasting and chronic high-fat feeding are associated with mitochondrial hyperacetylation [52, 53]. We speculate here that these stresses may dampen the activity of enzymes that protect against oxidative stress, such as SOD2 and IDH2, via direct acetylation [29, 54, 55]. If true, the subsequent ROS increase would promote gene expression to boost oxidative stress defense thus achieving a new hormetic steady state. In support of this notion, caloric restriction, fasting, and high-fat feeding initially spur an increase in SIRT3 transcription. Increased levels of SIRT3 could then deacetylate SOD2, IDH2, and several other mitochondrial proteins involved in redox regulation to minimize chronic ROS production. Interestingly, this response, while initially appropriate on a short-term high fat diet, is lost during chronic high fat diet feeding (>10 weeks) and associated with a downregulation of PGC-1α[53, 56]. As discussed above, the transcriptional increase of genes involved in mitochondrial energy metabolism and ROS detoxification during calorie restriction is strongly correlated with elevated expression of the transcriptional coactivator PGC-1α. This stress response may be sufficient to counter the mild stress of caloric restriction, but incapable of combating chronic high-fat feeding. When PGC-1α expression decreases, SIRT3 expression ultimately declines, oxidative stress worsens, and the fitness of the organism is diminished (Figure 4).

## PERSPECTIVE

While considerable more investigations are required, a causative role for oxidative stress in aging remains one of the most solid aging theories. The studies addressed in this review have demonstrated that sirtuins are intimately linked to the cellular response to oxidative stress. Moving forward, it will be important to develop experimental models in which the levels of oxidative stress and the activities of sirtuins can be precisely modulated to determine if sirtuins have a causative role in lifespan extension. Does mitochondrial sirtuin overexpression in mice extend lifespan? Do long-lived animals exhibit chronic low levels of oxidative stress? From a more practical standpoint, is it possible to rejuvenate tissue function by targeted overexpression of sirtuins to reduce oxidative stress? We look forward to future studies that will undoubtedly address many of these important questions.
